# Local Delivery of Therapeutics to the Cochlea Using Nanoparticles and Other Biomaterials

**DOI:** 10.3390/ph15091115

**Published:** 2022-09-07

**Authors:** Shreshtha Dash, Jian Zuo, Peter S. Steyger

**Affiliations:** Translational Hearing Center, Department of Biomedical Sciences, Creighton University School of Medicine, 2500 California Plaza, Omaha, NE 68178, USA

**Keywords:** drug delivery, blood–labyrinth barrier, otoprotective therapeutics, local delivery, cochlea

## Abstract

Hearing loss negatively impacts the well-being of millions of people worldwide. Systemic delivery of ototherapeutics has limited efficacy due to severe systemic side effects and the presence of the blood–labyrinth barrier that selectively limits or enables transfer of molecules between plasma and inner ear tissues and fluids. Local drug delivery into the middle and inner ear would be preferable for many newly emerging classes of drugs. Although the cochlea is a challenging target for drug delivery, recent technologies could provide a safe and efficacious delivery of ototherapeutics. Local drug delivery routes include topical delivery via the external auditory meatus, retroauricular, transtympanic, and intracochlear delivery. Many new drug delivery systems specifically for the inner ear are under development or undergoing clinical studies. Future studies into these systems may provide a means for extended delivery of drugs to preserve or restore hearing in patients with hearing disorders. This review outlines the anatomy of the (inner) ear, describes the various local delivery systems and routes, and various quantification methodologies to determine the pharmacokinetics of the drugs in the inner ear.

## 1. Introduction

The World Health Organization estimates that by 2050, over 700 million people, or one in every ten people, globally will experience disabling hearing loss [1]. Hearing loss negatively impacts personal well-being. Studies have shown that children and adults with hearing loss have a poorer quality of life due to reduced social interactions, isolation, a sense of exclusion, and depression [2], and in older people, this can lead to accelerated cognitive decline [3]. Hearing loss can result from many different types of inner ear disorders, including presbycusis (age-related hearing loss [ARHL]), genetic polymorphisms, trauma, exposure to noise, and ototoxic medications, to name a few. There are several therapeutic strategies to treat inner ear disorders, including systemic or local delivery of therapeutic agents, surgical intervention, and acoustic (e.g., hearing aids) and electric (cochlear implants) amplification [4]. This review specifically focuses on the local delivery of pharmacological agents for the management of inner ear disorders. We review various strategies used to deliver drugs to the inner ear and highlight their potential strengths and weaknesses.

Sensorineural hearing loss results from the dysfunction of the sensory and non-sensory cells, as well as neurons, present in the cochlea. Mammals, including humans, are unable to spontaneously regenerate many of these cell types. An active area of research is focused on preventing hearing loss due to treatment with cisplatin (and its derivatives) in anticancer chemotherapies, or aminoglycoside antibiotics for urgent treatment of severe bacterial infections [5]. Systemic delivery of ototherapeutics presumes that these drugs can reach their targets in their active form and at therapeutic concentrations. However, systemic administration of otoprotective drugs that achieve therapeutic concentrations in the inner ear often has limited efficacy due to unwanted systemic side effects from the high doses needed [6]. Hence, for otoprotective pharmacotherapies to be clinically relevant, it is imperative to develop safe and reliable drugs that can be delivered to the cochlea. In this review, we focus on (1) the anatomy of the ear, (2) delivery systems that target the cochlea, and (3) biomaterials that enable drug delivery to the cochlea. The safe and efficacious delivery of ototherapeutic agents that preserve or restore hearing will be a significant breakthrough in preventing permanent hearing loss.

## 2. Anatomy

**Figure 1:**
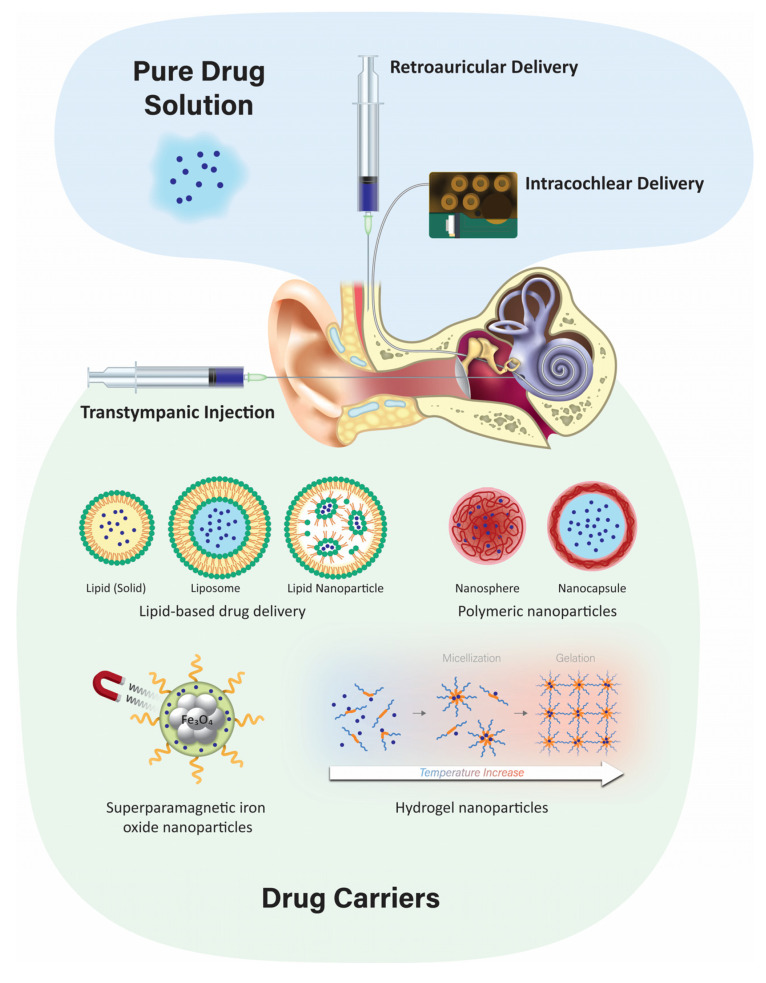
A schematic summary of various drug delivery routes and methodologies to locally deliver therapeutics to the inner ear. Not to scale.

**Figure 2:**
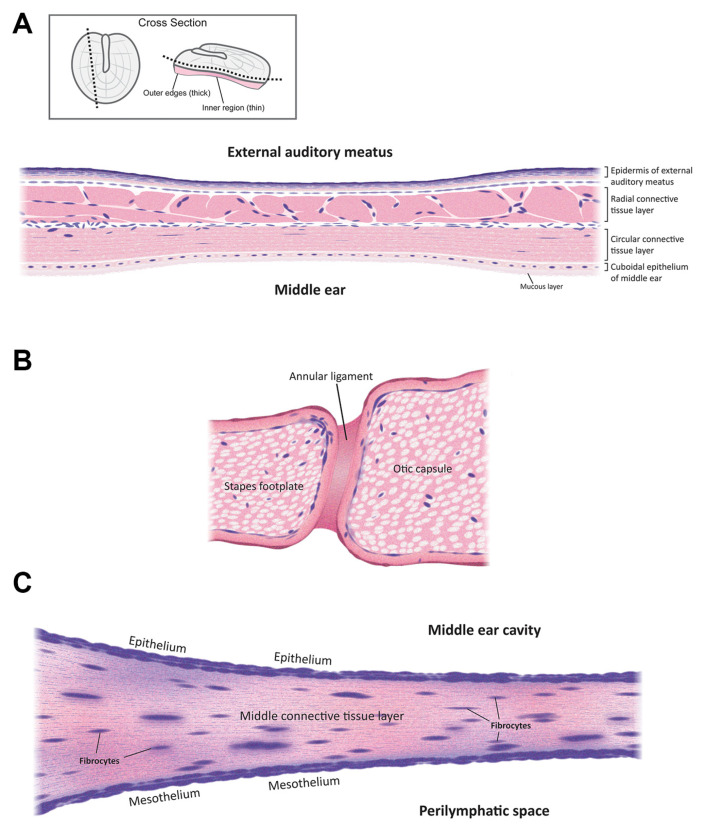
Schematic cross-sections of (**A**) the tympanic membrane; (**B**) the oval window membrane; and (**C**) the round window membrane. Not to scale.

The mammalian ear is a complex sensory organ critical to hearing and maintaining balance (Figure 1). The outer ear includes an external auditory meatus (ear canal) that is slightly curved [7]. The outer and middle ear are separated by the semitransparent tympanic membrane that is thinnest in the center (50–70 µm) and thickest (~100–120 µm) near the peripheral rim [8]. This membrane is concave, with its deepest point projecting into the middle ear cavity. The outer and innermost layers of the tympanic membrane are extensions of the epidermis lining the external auditory meatus and the mucosal epithelial layer lining the middle ear cavity. The middle, extracellular layer of the tympanic membrane (Figure 2A) is formed of radial and circular connective tissue comprised of collagen and elastic fibers and innervated by several cranial nerves [9].

The middle ear is a narrow, air-filled space with an opening in the anterior wall that leads to the nasopharynx—the Eustachian tube (Figure 1). The medial wall separates the middle ear from the inner ear and has two small openings: the oval window and the round window. These are considered the main passage routes for drugs entering the inner ear from the middle ear. There are three middle ear bones (ossicles), the malleus, incus, and stapes, that link the tympanic membrane with the cochlea. This ossicular chain acts as an impedance transformer, converting acoustic air pressure waves into fluid pressure waves at the oval window [7,10].

The cavity of the oval window is closed by the ossified footplate of the stapes, which is attached to the otic capsule by the annular ligament (Figure 2B) [11]. The average dimension of the oval window in humans is 1.26 mm at its narrowest and 2.40 mm at its widest [12]. The oval window has diverse neurovascular structures such as the ptotic facial nerve and the persistent stapedial artery. The oval window is medially bounded by the vestibule [11].

The round window is covered by a thin membrane known as the round window membrane (RWM; Figure 2C). The RWM is made up of a single layer of epithelial cells, with numerous cylindrical-shaped mitochondria, rough endoplasmic reticulum, a Golgi complex, and sparse microvilli in the middle ear cavity. The inner mesothelial cell layer of the RWM faces the perilymphatic space and contains ‘gaps’ between the epithelial cells, allowing direct contact of the intervening connective tissue with perilymph in the cochlear scala tympani [13]. The middle layer of the RWM is composed of connective tissue [14] that contains fibroblasts, collagen, and elastic fibers as well as blood and lymph vessels [13]. The average thickness of RWM in humans is ~70 µm, with the edge being thicker than the center [15].

The stapes and oval window, along with the RWM, serve as a physical barrier that protects the inner ear [16]. Locally-administered drugs in the middle ear can pass through the oval or round window membranes to reach the perilymph enclosed within the scala vestibuli or tympani, respectively [17]. The permeability of these membranes is dependent on drug charge, size, concentration, configuration, liposolubility, and thickness [18].

The inner ear consists of a bony labyrinth within which are soft tissues and several ducts, collectively known as the membranous labyrinth. This membranous labyrinth can be subdivided into the vestibular labyrinth and the cochlear labyrinth. The vestibular labyrinth contains utricle and saccule and three semicircular canals. The vestibular organ detects gravity, acceleration, motion, and rotation and is known as the organ of balance [7]. The cochlear duct is a snail-shaped, membranous labyrinth that is fluid-filled and contains the sensory epithelium known as the organ of Corti that senses sound [19]. The fluid spaces surrounding the cochlear duct (scala media) are known as the scala vestibuli and scala tympani and are filled with perilymph, while the scala media is filled with endolymph [20]. Tight junction-coupled epithelial cells (of several different lineages) line these fluid-filled scalae [21]. Perilymph and endolymph differ in their ionic composition, with perilymph having 5 mM K^+^ (typical of extracellular fluid) and, uniquely in the mammalian body, endolymph consisting of 150 mM K^+^ [22]. The organ of Corti is arranged on the endolymph-facing surface of the basilar membrane with orderly, longitudinal rows of sensory hair cells known as inner and outer hair cells, each entirely surrounded by tight junction-coupled supporting cells [23].

## 3. Delivery Routes

### 3.1. Systemic Delivery

Systemic delivery of therapeutics to the inner ear typically involves delivery via the vasculature and crossing the tight junction-coupled blood–labyrinth barrier (BLB). The BLB is fundamentally similar to the blood–brain barrier (BBB) and is well described elsewhere [24]. Paracellular flux across the BLB is not thought to occur under normal physiological conditions but may arise during inflammation, as for the BBB [25]. Drug transport across the BLB could involve similar mechanisms to that across the BBB [24,25,26]. These include (i) diffusion of lipophilic molecules (e.g., solvents) across cellular membranes; (ii) transcellular flux of hydrophilic drugs (e.g., aminoglycosides) via permeation of non-selective cation channels [27,28]; translocation via substrate transporters [29]; or transcytosis through the cell [26].

The systemic route of drug delivery has several limitations, including systemic side effects, e.g., kidney failure, neuromuscular blockade, as well as hearing loss in some cases [30]. The variable incidence of efficacy within the inner ear (or ototoxicity) can be due to the inconsistent penetration of the BLB. Local delivery of drugs to the ear can avoid (some of) these limitations and has become a standard treatment technique for treating Meniere’s Disease or other vestibular disorders, such as vertigo, by partial or total ablation of vestibular sensory cells [31]. Below, we briefly discuss several types of local delivery systems to the inner ear.

### 3.2. Topical Delivery via the External Auditory Meatus

Topical delivery of drugs to the ear canal is used to treat inflammation of the outer and middle ear. Antibacterial and antifungal ointments (or drops) are applied directly to the ear canal up to three times a day. The advantages of this administration route are that it is inexpensive, and it can be performed as an outpatient procedure or at home by the patient. There are a few disadvantages, including apparently limited therapeutic application, as it cannot currently be used for drug delivery to the inner ear as the tympanic and round window membranes are thought to act as barriers, as well as loss of drug from the middle ear via the Eustachian tube [32]. However, ototoxic drugs like gentamicin are not used clinically as ear drops as they can cause serious side effects such as hearing loss and tinnitus when used by patients with a perforated tympanic membrane [33], suggesting that topically-applied therapeutics can reach the inner ear. Moreover, concerns exist regarding the efficacy of topically-administered drugs against bacteria that have infected the middle ear and mastoid cavities [34]. Additionally, topical administration typically requires the patient’s reliability and compliance [32].

### 3.3. Transtympanic Delivery

It is a minimally-invasive injection that is typically performed in a clinic setting. The major advantage of transtympanic delivery is that it provides a potential direct route of administration to the inner ear. Typically, transtympanic delivery of drugs is injected onto the round window (and round window niche) and relies on the permeation of compounds through the RWM into the perilymphatic scala tympani in the cochlea. Permeation of the RWM is dependent on various factors, including the drug size, charge, lipophilicity, concentration, and formulation of the substances [18]. Following administration to the middle ear, local absorption takes place via the round window and the oval window into the cochlear perilymph and subsequent diffusion into the vestibular system. Previous studies have demonstrated that prolonging the contact duration of the drug formulation with the RWM can increase drug levels in the inner ear, but the pharmacokinetics for individual drugs remain complex to interpret [35]. Permeation of drugs across the round window membrane depends on passive diffusion and active transport [31]. Manipulations of the RWM have been shown to increase the entry of drugs following transtympanic administration. A variety of agents have been screened that are used to improve drug penetration in other biological systems for their capacity to increase entry into the inner ear. Some of these agents include benzyl alcohol, dimethyl sulfoxide, saponin, caprate, and Poloxamer 407. Benzyl alcohol is commonly used as a preservative in drug formulations and has been found to be the most effective permeabilizing agent [36]. In preclinical settings, some rodents are known to have false RWM (as well as patent RWM), increasing the variability of such studies. The presence of false RWMs was found to be present in ~25% of patients with Meniere’s disease compared to 5% of patients without Meniere’s disease [37].

Until recently, delivery of therapeutics to the RWM was considered the primary entry route into the inner ear. Yet, greater loss of vestibular function and utricular hair cells was observed when gentamicin was delivered directly to the stapes footplate of the oval window compared to RWM delivery. In addition, greater high-frequency hearing loss as well as loss of basal cochlear hair cells were also observed after delivery to the oval window. These data suggest that capillarity enables a flux of gentamicin from the RWM into the scala vestibuli via the stapes footplate to account for the efficacy of gentamicin suppression of symptoms of Meniere’s disease [38,39]. To validate whether the basal outer hair cells in humans are also more at risk from oval window delivery than RWM delivery (in humans as in preclinical models), extended high-frequency audiometry will be required.

### 3.4. Retroauricular Delivery

Microcomputed tomography scanning of human temporal bone has revealed multiple large air-filled tuberculae, divided by bony septae. These air-filled spaces known as retroauricular microchannels are located behind the posterior wall of the ear canal and are rich in blood supply [40]. These microchannels have been utilized to deliver drugs to the middle or inner ear via parenteral retroauricular injections [41]. A study in a group of 20 healthy adults given a retroauricular injection of adrenaline found a significant drop in pressure in the middle ear cavity compared to the control group. It is thought that these microchannels enabled the adrenaline to enter the mastoid mucosa to induce vascular constriction and mucosal decongestion, leading to decreased middle ear pressure [42]. In another study, patients were administered glucocorticoids orally or by retroauricular or transtympanic injection. Higher visual analogue scale (VAS) pain scores and sleeping disorders were recorded in patients receiving transtympanic injections compared to retroauricular injections. However, no significant difference in therapeutic efficacy (i.e., hearing improvement) was noted among the three groups [43]. More recently, the distribution of dexamethasone in the inner ear following postauricular or intramuscular injection by in vivo optical imaging was examined. The local fluorescent intensity in the inner ear after postauricular injection was higher than that by intramuscular injection. There was a sustained release effect after postauricular injection, and drugs administered by this route might enter the endolymphatic sac via the posterior auricular artery and occipital artery [44].

### 3.5. Intracochlear Application

Intracochlear delivery bypasses the tympanic, oval, and round window membranes by infusing drugs directly into perilymph within the cochlea, presumptively providing greater control over drug concentrations within the cochlea [14]. Intracochlear injection through the RWM provides higher and more sustained drug levels compared to transtympanic injection onto the RWM [16]. However, the perforation of the RWM caused by the needle can release inner ear pressure, allowing cerebrospinal fluid (CSF) to enter the scala tympani via the cochlear aqueduct [45].

Other intracochlear drug delivery methods include infusion via an osmotic minipump, or a catheter built into the electrode array of a cochlear implant with an implantable peristaltic pump attached to it [18,46]. The osmotic pump is implanted subcutaneously with a cannula threaded into the middle ear cavity and inserted through the RWM or through the bone directly into the cochlea via a cochleostomy. These pumps can provide continuous infusion of drugs for up to 6 weeks [47]. Osmotic pump delivery of betamethasone to treat a vestibular disorder in guinea pigs showed a shorter duration of recovery than in controls of untreated with no osmotic pump and saline delivered through osmotic pump groups [48]. Osmotic pump delivery of an anti-apoptotic agent, Z-VAD-FMK, infused into the cochlea of guinea pigs for 14 days revealed less noise-induced hearing loss and lower hair cell loss compared to noise-exposed, untreated ears [49]. However, osmotic pumps are limited in that drug delivery cannot be stopped, nor can the flow rate be changed once the pump is started, and the volume of vehicle plus drug infused over time can exceed the volume of perilymph in the inner ear [47]. Recently, a micropump with a better automated control of drugs for intracochlear delivery at safe and slower flow rates without increasing the volume of perilymph in the cochlea has been described [50].

Surgical implants, including cochlear implants, can rehabilitate severe-to-profound sensorineural hearing loss. Recently, intracochlear controlled release drug delivery in combination with cochlear implants has been developed. This can improve the performance of cochlear implants by preventing fibrosis induced by insertion trauma, protecting the neuronal structures and by providing controlled drug release [51]. However, potential limitations include an enhanced risk of infection, particularly meningitis, associated with the surgery [52]; damage to the facial nerve; and loss of residual hearing. An implantable peristaltic pump connected to a cochlear implant electrode array has been developed for long-term delivery and effective dose-control in non-human primates (macaques). The infusion time ranged from 2–24 h to reach maximum peak concentrations, demonstrating feasibility [46].

## 4. Localized Inner Ear Delivery Methods

Localized drug delivery to the cochlea has the advantages of targeted delivery with higher bioavailability and minimal systemic side effects. The ideal delivery system would be a carrier system that delivers pharmacotherapeutics across the intact tympanic, oval, and/or round window membranes to efficaciously treat inner ear disorders.

Before a carrier system is chosen, advanced knowledge of the preferred drug and its physicochemical properties is required to determine the appropriate formulation and delivery method. In the field of drug discovery, Lipinski’s *rule of five* is frequently used to predict the absorption and solubility properties of a drug [53,54], and includes molecular weight, lipophilicity, polar surface area, hydrogen bonding, and charge [55]. These properties assist in the design and screening of new candidate drugs by predicting if a chemical compound has the appropriate bioavailability and pharmacokinetics. The rule states that ideal candidate drugs will have a logP ≤ 5 (the partition coefficient of a molecule between aqueous and lipophilic phases [usually water and octanol]), a molecular mass ≤500, ≤10 hydrogen bond acceptors, and ≤5 hydrogen bond donors. Molecules that fail to follow one or more of these rules may have difficulty with bioavailability [56].

Given the uncertainty of how drugs are metabolized in the inner ear, it is paramount to determine the physicochemical properties of candidate drugs for inner ear delivery. These may differ from the well-established Lipinski *rule of five* for systemic drug development. Thus far, much of our knowledge of these physicochemical properties for inner ear delivery are based on a few empirical studies of selected agents such as local anesthetics, corticosteroids (e.g., dexamethasone), monoclonal antibodies, growth factors, apoptosis inhibitors, and vectors for gene therapy under clinical investigation [57,58]. Many more studies are required to establish the optimal physicochemical properties for efficacious inner ear therapeutics, e.g., the ability to cross the blood–labyrinth barrier, the tympanic, oval, or round window membranes, as well as intracochlear epithelial barriers.

### 4.1. Developing Different Injectable Solutions like Hydrogels

Developing biomaterials that are non-ototoxic and deliver therapeutics that can cross inner ear-specific biological barriers, e.g., the RWM barrier, is a significant challenge for the efficient local delivery of therapeutics to the inner ear, avoiding the side-effects associated with high doses of systemically-administered drugs [59]. However, local delivery methods have their own challenges, such as clearance of the drug via the Eustachian tube and variable diffusion through the RWM. These challenges can be overcome by developing a sustained drug release system that can prolong direct drug contact with the RWM for effective delivery [60].

Recent studies have explored injectable hydrogel drug formulations that can improve drug contact with the RWM [16]. These hydrogels are fluid-like at room temperature and quickly gelate at body temperature to promote the sustained release of encapsulated drugs, increasing drug contact time with the RWM [61]. Hydrogels have numerous advantages, including increased biocompatibility, adjustable biodegradability, low toxicity, and good swelling behavior [62]. The degree of swelling is a critical parameter, with a higher concentration of polymers leading to greater swelling and slower drug release [63]. In the middle ear, hydrogels are typically injected near the RWM and RWM niche. This enables prolonged diffusion of the released drug across the RWM at appropriate therapeutic concentrations [64]. Hydrogels have been developed in several formulations for inner ear drug delivery, including polymers such as chitosan or PEG-based hydrogel, Poloxamer 407, and hyaluronic acid [65,66,67,68]. Injectable PEG-based hydrogel has been shown to be an effective and safe method for inner ear delivery. In guinea pigs, the dexamethasone concentrations in perilymph were maintained for at least 10 days for the PEG hydrogel as compared to the control sample of free dexamethasone [69]. Hyaluronic acid, when applied to the RWM of guinea pigs prior to delivering an adenovirus, provides an atraumatic and feasible method of delivering transgene into the inner ear [70].

### 4.2. Poloxamer 407 and Its Mechanism

Poloxamer 407, also known as Pluronic^®^ F-127, is the primary polymer for formulating hydrogels used for inner ear delivery of therapeutics [59]. Poloxamer 407 is an amphiphilic, non-ionic triblock copolymer consisting of a residue of polyoxypropylene (POP) between two units of polyoxyethylene (POE). It is a widely used thermo-sensitive hydrogel due to its non-irritating action on biological membranes and can remain as a gel for several weeks to months [71]. Its thermo-sensitivity is due to the hydrophobic interactions between the copolymer chains of Poloxamer 407. As temperature increases, copolymer chains of Poloxamer 407 aggregate to form a micellar structure due to the dehydration of hydrophobic poly(propylene oxide) units [72] with a micelle diameter in the 20–100 nm range. The hydrophobic core is the drug loading site, creating space for the encapsulation of drugs through chemical interactions. The properties of the inner and outer shell determine the rate of drug release. Different methods are employed for encapsulating drugs in Poloxamer 407, such as direct dissolution, evaporation, and freeze-drying [73]. The gelation of Poloxamer 407 is reversible once gelling conditions, such as temperature, pH, or chemical, are removed [62].

In guinea pigs, a single intratympanic dose of dexamethasone-Poloxamer 407 can extend the duration of the drug in the perilymph to 10 days [74]. Similarly, N-acetylcysteine (NAC), a thiol-containing antioxidant with otoprotective effects in preclinical models of cisplatin-induced hearing loss [75], can increase and sustain the release of NAC in cochlear perilymph [67]. 

### 4.3. Nanoparticulate Injection Systems

Recently, there has been a rapid development in several nanoparticulate-based drug delivery systems (Table 1), although challenges still need to be resolved [76]. Nanoparticulate drug delivery is one of the most advanced technologies in drug design due to its advantages such as surface modification, improved drug solubility, stability, and bioavailability, as well as sustained controlled drug release at the target site. Injecting nanoparticulates at the targeted site leads to lower systemic toxicity, fewer side effects, improved kinetics of the drug, and extended drug bioavailability [77]. There are two primary nanoparticulate strategies: passive and self-delivery. In passive delivery, drugs are encapsulated in nanocarriers and are slowly released from the carriers. In self-delivery, drugs are conjugated to the carrier for easy delivery, and the drug dissociates from the carrier quickly at the presumptive targeted site, e.g., in the vicinity of tumors [78,79]. A large variety of nanoparticles have emerged, including polymeric, liposomes, and lipid-based structures, among others. Below, we briefly discuss several types of nanoparticles.

#### 4.3.1. Polymeric Nanoparticles

Biodegradable polymeric microparticles or nanoparticles have been developed for a wide range of therapeutic applications and as inner ear drug delivery systems. They are often based on poly (lactic) co-glycolic acid (PLGA) or chitosan [6] and have advantages over other delivery systems due to their biocompatibility, biodegradability, small size, long shelf life, stability during storage, and highly reproducible formulation methods [59]. Nanoparticles have a diameter of <1 µm and are usually formulated with diameters of 100–300 nm, and for inner ear delivery, ~200 nm or less [4]. Polymeric nanoparticles can also incorporate visualization agents such as fluorescent dyes and MRI contrast agents [100]. Iron oxide nanoparticles have been extensively studied as a contrast agent for MRI. It has a magnetic core and different ligands focus on targeting specific sites or cells. PLGA is one of the more popular polymeric particles that can encapsulate both hydrophobic and hydrophilic drugs for intratympanic delivery and can be transported across the RWM into perilymph via the transcellular pathway [101].

#### 4.3.2. Solid Lipid Nanoparticles

Solid lipid nanoparticles (SLNs) are a novel class of stable nanoparticles that are particularly suitable for the encapsulation of hydrophobic drugs such as curcumin, resveratrol, or quercetin [102]. They can also act as a carrier for hydrophilic drugs when formulated without the use of organic solvents [103]. SLNs are composed of a hydrophobic triglyceride core with an amphiphilic surfactant shell [104]. SLNs are biodegradable, biocompatible, and are non-ototoxic over a wide dose range. Low doses of hydrocortisone encapsulated in SLNs increase their protective effect and prolong the survival of auditory cells treated with cisplatin in vitro [97]. In vivo application of SLNs has shown no interference in hearing threshold or loss of hair cells [105].

#### 4.3.3. Liposomes

Liposomes are composed of two layers of amphipathic molecules with a hydrophilic external layer and an internal surface composed of a phospholipid bilayer [106]. A key advantage of using liposomes for cochlear drug delivery is their ability to control drug release [96]. In an in vivo study, the microinjection of cationic liposome-mediated gene transfer into guinea pig cochleas revealed that transgene expression was steady for up to 14 days in the neurosensory epithelia and surrounding tissues without any toxicity [107]. Others have successfully demonstrated cell–gene delivery of therapeutic agents to the inner ear using a liposome-mediated delivery method [108].

#### 4.3.4. Superparamagnetic Iron Oxide Nanoparticles (SPION)

SPIONs are Fe_3_O_4_ particles that can be magnetically controlled to focus on the migration of particles into the inner ear after crossing the RWM [60]. These particles are encapsulated in a polymeric layer of PLGA [109] or chitosan [110] to contain the therapeutic agent. Other polymers such as polyacrylic acid and polyvinylpyrrolidone are used to form a coat around iron oxide nanoparticles to increase stability and improve their magnetic properties [111]. This technique has been demonstrated in vivo in rat and guinea pig models, as well as in vitro in cell lines and the human temporal bone. Biocompatibility and safety have been demonstrated by various methods, including hair cell survival in organotypic cell cultures [112].

### 4.4. Advantages and Disadvantages of the Nanoparticulate Injection System

Nanoparticles are created from a variety of materials with a diameter range of 10 to several 100 nanometers, and customized to encapsulate various therapeutic agents [113]. Nanoparticles are widely used for non-invasive application, sustained, or controlled release of drugs, drug stabilization, and surface modification for targeting specific organs [114]. Various studies that administered nanoparticles onto the RWM have shown successful delivery of biomaterials into the inner ear [115]. Nanoparticles can enter the perilymph and the endolymph [106] following intratympanic administration and can be targeted to a specific intracochlear site of interest. Nanoparticles, when combined with hydrogel, improve the bioavailability of the drug at the targeted site and prevent rapid drug release [106]. Nanoparticles can also be conjugated to peptides that can penetrate cells, or their surface can be modified to increase their contact with RWM [116]. Challenges for nanoparticle delivery into the inner ear include limited access to the inner ear and poor uptake of drugs by inner ear cells [106]. Major disadvantages of liposomes include low encapsulation efficiency for lipophilic drugs, and if their formulation requires organic solvents, the marketed product might be leaky and unstable in biological or aqueous fluids [103]. The reduced biodegradability of SPIONs coupled with their side effects, such as inflammation, generation of reactive oxygen species, genotoxicity [117], and higher time of residence in the cochlea, limits their use in humans [118]. The use of nanoparticles to achieve greater drug distribution in the cochlea has been promising, but adverse effects from the drugs or biomaterials within the inner ear remain a concern [14].

### 4.5. Positively-Charged Biomaterials for Local Drug Delivery

The charge of nanoparticles can determine their uptake by inner ear hair cells and their penetration of epithelial membranes. Phospholipid-based nanoparticles with a positive charge of +26 mV were taken up by sensory hair cells at a two-fold higher rate than nanoparticles with a neutral or negative charge. This is likely due to the interaction between positively-charged nanoparticles and negatively-charged lipid plasma membranes [119]. The addition of cationic polyethylene glycol (PEG) to phospholipid-based nanoparticles increases their trafficking across the RWM but has enhanced cytotoxicity [120]. Nanoparticulates containing cationic-PEG to deliver dexamethasone to the RWM in mice provide an anti-inflammatory effect during combined kanamycin and furosemide treatment and higher cellular uptake within the organ of Corti [99]. Positively-charged chitosan nanoparticles enter the inner ear at a faster rate [59]. Positively-charged chitosan nanoparticles containing D-glucosamine and N-acetyl-D-glucosamine facilitate penetration of lipid cell membranes in the inner ear [119]. Other positively-charged nanoparticles, such as 1,2-dioleoyl-3-trimethylammonium-propane (DOTAP), are distributed widely in the inner ear after RWM application [14]. In rodents, positively-charged ferritin readily passes through RWM, while negatively-charged ferritin does not [13]. Cationic carriers, or cationic drugs conjugated to carriers, can effectively interact with negatively-charged membranes, enabling multilevel drug targeting at tissue and cellular levels. These carriers penetrate tissues and cells rapidly and in high concentrations due to electrostatic interactions with negatively charged glycosaminoglycans [121].

#### Advantages and Disadvantages of Positively-Charged Biomaterials

In rodents, cationic ferritin easily passes through the RWM, whereas anionic ferritin does not [122]. The negatively-charged glycocalyx and the anionic phospholipid bilayer of cell membranes act as barriers to cellular entry by repelling negatively-charged, hydrophobic or large molecular weight molecules, leading to poor uptake of anionic therapeutics [123]. Cationic biomolecules utilize electrostatic interactions to overcome the barrier formed by the anionic bilayers of the cell membrane to facilitate entry of cationic therapeutics into target cells via direct penetration, permeation of selected ion channels, or endocytosis [124]. A positively-charged moiety, such as a cell-penetrating peptide, can be added to nanoparticles to improve their permeability into cells or for gene transfer [81]. 

Despite having several advantages, the biological potency of cationic biomaterials and their side-effects remain largely unknown [121]. Recent in vivo studies show that high doses of cationic liposomes and polymeric nanoparticles interact with Na^+^/K^+^-ATPase cationic binding sites, resulting in cytoplasmic swelling, acute cellular necrosis, and leakage of mitochondrial DNA. This triggers molecular disruption and severe inflammatory responses via TLR9 and MyD88 signaling [125]. When highly-positive-charged biomaterials enter cells, they electrostatically interact with negatively-charged cytoplasmic proteins that then precipitate as large clusters and can destabilize the cell membrane, causing toxicity [126]. Positively-charged nanoparticles can be ototoxic due to their low biodegradability, increased production of intracellular reactive oxygen species, and cell membrane damage, limiting their use in treating inner ear disorders [127].

### 4.6. Negatively-Charged Biomaterials for Local Drug Delivery

Negatively charged polymers have been frequently used for the preparation of nanoparticles due to their biocompatible properties. PLGA, an anionic polymer, is one of the most successful biodegradable systems for inner ear drug delivery [128]. PLGA with penetration enhancers, such as cell-penetrating peptides, has been used to investigate their impact on cochlear drug delivery in vivo [129]. PLGA nanoparticles coated with Poloxamer-407, with a zeta-potential of −15.90 mV, had a 1.6-fold higher distribution in the cochlea as compared to anionic PLGA nanoparticles coated with chitosan [128]. Negatively charged nanoparticles, with a surface charge of −22.1 mV, were administered intratympanically, diffused across the RWM, and distributed in the basal and middle cochlear turns when visualized by transmission electron microscopy [130]. Negatively-charged gelatin hydrogels composed of highly biocompatible polymers are used for controlled drug release, such as insulin-like growth factor 1 to the inner ear after noise-induced hearing loss in guinea pigs, resulting in increased outer hair cell survival [131]. Zeolitic imidazole nanoparticles have great potential to deliver drugs, proteins, and RNA to the inner ear for the treatment of noise-induced hearing loss. These nanoparticles are anionic carriers with superior cell viability and biocompatibility [132]. 

#### Advantages and Disadvantages of Negatively-Charged Biomaterials

In vitro assays of negatively charged nanoparticles show higher cell survival and viability than assays with positively charged nanoparticles. The higher diffusibility of positively-charged drug delivery systems can lead to greater drug deposition in the inner ear, while slightly negatively-charged nanoparticles have higher potential to be used in inner ear therapy due to reduced nanoparticle aggregation and inadvertent interactions with (serum or other extraceullar) proteins [133]. The positive charge across the lumenal endothelial membrane resists the passage of positively-charged molecules and facilitates the entry of negatively-charged molecules between the vascular system and the intra-strial space [134]. In rodents, anionic nanoparticles had significantly higher efficiency for hearing protection from noise-induced trauma [132]. Nonetheless, negatively-charged biomaterials have certain drawbacks, including lower bioavailability in cochlear perilymph due to poor diffusion through the RWM, making these biomaterials less ideal. Moreover, this lower bioavailability limits targeted drug delivery by anionic carriers [128].

## 5. Pharmacokinetics and Pharmacodynamics of the Drugs in the Inner Ear

Pharmacokinetics describes the movement of drugs within the body. It includes absorption, distribution, metabolism, and excretion (ADME), as described briefly below. The pharmacokinetics of locally-applied drugs within the inner ear are complex due to the interaction of many factors, such as passage of the drug through the RWM, distribution in perilymph, endolymph, various tissue compartments of the ear, and clearance into CSF via the cochlear aqueduct [135]. 

**Absorption** is the diffusion of drugs from the middle ear into the perilymph via the RWM, OWM, or through the cochlear bony shell [136]. Absorption after systemic application includes trafficking of the drug from the vascular system into the inner ear across the blood–labyrinth barrier [137].

**Distribution** descriptively details the intracochlear spread of therapeutics in the perilymph, the endolymph, and tissues of the inner ear. It depends on various factors, such as drug permeability, lipid solubility, blood flow, etc. Distribution is also concentration gradient-dependent [135]. 

**Metabolism** refers to the chemical conversion of drugs into metabolites that are more bioactive, particularly in the liver [137]. Dexamethasone phosphate, a prodrug which is commonly injected transtympanically, is converted into an active form after absorption into the inner ear [138].

**Elimination** is the clearance of the drug or its metabolites from the body [135]. In the inner ear, drug or their metabolites can be eliminated from the cochlear fluid into cochlear cells or into the blood and CSF as well as into the middle ear [135]. From the middle ear, it can be eliminated via the Eustachian tube into the nasopharynx [137]. The higher the drug viscosity, the slower the excretion through the Eustachian tube. Drug concentrations can also decline due to structural breakdown or metabolism [135].

The concept of drug **Liberation** was added to ADME and, in 2009, applied to the ear by [139]. Liberation represents the release of a drug from its carrier into the targeted area. The formulation could be a polymer-based gel or nanoparticles, liposomes, etc. It could also be drops applied to the external ear or fluid injection given via transtympanic or intracochlear routes. Drug formulations play an important role in influencing liberation. Other important aspects are application procedures or devices, etc. that can influence liberation [135]. In the following section, we have described the cochlear pharmacokinetics of various drugs and the methodologies used to quantify these drugs in the inner ear.

Sometimes the **Toxicity** of a compound is also considered, yielding ADME-Tox, ADMET, or LADMET. Toxicity is an essential factor that affects a new molecule’s potential to become a drug. Appropriate ADME-Tox or ADMET properties at therapeutic doses are essential for drug discovery [140]. Parameters used to characterize toxicity include the median lethal dose (LD_50_) and the therapeutic index [141].

## 6. Quantification of Drugs in the Inner Ear

Drug concentrations in perilymph or endolymph collected from the cochlea are typically analyzed with high performance liquid chromatography (HPLC) [142]. Different administration routes have shown different pharmacokinetics, such as that the level of corticosteroids in perilymph was significantly higher after transtympanic injection compared to systemic administration [143]. In guinea pigs treated with dexamethasone, drug levels in the basal region exceeded those in the apical region [144]. When guinea pigs were treated with cisplatin systemically, its concentration in perilymph was found to be 4 times higher in the base than in the apex [145]. In most species, the endolymph has much smaller volume than the perilymph (~2 µL compared to ~10 µL, respectively, in guinea pigs) and is more difficult to access [142]. However, the endolymph levels of corticosteroids have been analyzed quantitatively in the guinea pig cochlea and a higher level of the drug was found in the endolymph than in the perilymph at the 1-h mark after transtympanic dosing [143].

Liquid chromatography coupled with mass spectrometry (LC-MS) has the additional advantage of highly sensitive detection of therapeutic agents and accurate separation of compounds from biological samples [142]. The perilymph in guinea pigs was analyzed in LC-MS to understand the pharmacokinetics of dexamethasone administered via intraperitoneal, transtympanic, and postaural injections. Transtympanic administration of dexamethasone resulted in its higher concentration in the perilymph and cochlear tissues compared with the other delivery routes. A decreasing basal to apical gradient of dexamethasone uptake was also found in the cochlea after transtympanic administration, but not with the other administration methods [146]. In an in vivo study, d-methionine, an antioxidant, was administered intravenously, orally, intranasally, or intratracheally, and perilymph as well as endolymph were sampled and analyzed by LC-MS. The highest concentration of the antioxidant was seen following intratracheal delivery. This unexpected finding is thought to be due to the enormous surface area of the lungs, coupled with low enzymatic activity, enabling direct absorption of inhaled compounds into plasma [147].

Immunoassays are employed for drug quantification in both preclinical research and clinical tests. Its advantages are high sensitivity and specificity and, easy handling with time and cost savings [148]. However, when compared to HPLC and LC-MS, its sensitivity and selectivity are lower. Moreover, immunoassays can only be used for drugs/antigens for which antibodies have been developed. In the guinea pig cochlea, when gentamicin was administered from the round window membrane, decreasing concentrations along the longitudinal cochlear axis were found. The concentration of gentamicin was >4000 times higher at the base than at the apex [149]. After systemic administration of gentamicin via intravenous or subcutaneous routes, gentamicin levels in perilymph were higher at the apex and gradually decreased towards the basal region [150].

Fluorescence imaging using confocal microscopy is widely utilized to analyze biological phenomena. It provides true three-dimensional (3D) optical resolution by blocking the fluorescent signal outside the focal plane. It is also characterized by high spatial resolution of (several) hundred nanometers and can also determine the cellular and subcellular localization of therapeutic agents in biological samples [142]. Dexamethasone injected via the transtympanic route was readily immunolocalized in the hair cells at 12 h, whereas systemically-administered dexamethasone was only weakly immunolocalized at 6 h, suggestive of less uptake or more rapid elimination from hair cells. Moreover, after transtympanic administration, dexamethasone was retained in cochlear tissue for at least 7 days [119]. The distribution and pharmacokinetics of gentamicin were similarly analyzed in chinchillas. The difference in the distribution of gentamicin between transtympanic administration and a sustained release from the osmotic pump was analyzed. The fluorescent signal was concentrated on the spiral ganglion, lateral wall, and organ of Corti. No differences were seen between the staining patterns of the different methods of gentamicin administration [151]. This approach has a few disadvantages, such as only single timepoint data can be obtained from an individual animal, fixation agents perfused into the cochlea may wash out the drug/antigen, photobleaching might occur due to high-power laser pulses, and fluorescence imaging is relatively less sensitive quantitively compared to HPLC and MS. In addition, fluorescent tags on conjugated molecules can change the pharmacokinetics and distribution properties of the native drugs [152].

Imaging mass spectrometry is an advanced technique that extends mass spectrometry to microscopic imaging capabilities. Recently, major progress has been made in matrix-assisted laser desorption/ionization (MALDI) imaging of biological samples. It can demonstrate the subcellular distribution of drugs and biomolecules in the sample and the localization of proteins in tumors [153]. Spatial resolution is also challenging, and only a few instruments have high spatial resolution sufficient for distinguishing the heterogenous cell types within the bone-encapsulated cochlea.

Electrochemistry is an analytical method for detecting substances in vivo. A prepared electrode is exposed to a solution of drug(s). A suitable potential is applied to this sensor, which causes a redox reaction of the drug and releases electrons whose number is proportional to the concentration of the therapeutic agent. Therefore, the detected current corresponds with the concentration of the drug [154]. The electrode is constructed of a gold-, platinum-, or boron-doped diamond. Its major use is in tracking neurotransmitters in the brain [155]. Ascorbate concentrations in perilymph sharply decreased within minutes when salicylate was injected into the perilymph of guinea pigs when measured by using carbon fiber microelectrodes with multiwalled carbon nanotubes [156]. Limitations of this procedure include invasive surgical procedures to expose the cochlea and that certain types of chemical compounds are electrochemically inactive [142]. 

## 7. Conclusions

The recent focus on optimizing local delivery of therapeutics to the inner ear has improved our understanding of how drugs can be delivered to a complex and inaccessible organ such as the inner ear. Regardless of the route of administration, designing drug delivery systems to target the cochlea to preserve, protect, and restore hearing faces numerous challenges. The complexity of the anatomy of the inner ear with its labyrinthine endothelial barrier (the BLB), its relatively inaccessible location, and its small size complicate the assessment of drug delivery, distribution, and clearance. Since cochlear fluids are only present in very low volumes, it is difficult to quantify drug concentrations in these fluids without contamination by other fluids such as CSF.

Local delivery has the advantage of maximizing targeted effects in the inner ear while minimizing systemic toxicity. Intratympanic injections are a relatively minimally invasive procedure. Future research to enhance permeation through the RWM and methods to increase the release duration of therapeutic agents from biomaterials may provide higher and more sustained concentrations of the drug in the cochlea following intratympanic administration. The use of nanoparticles encapsulating therapeutic agents that can target the sensory hair cells in the inner ear is innovative and exciting.

Currently, there are no FDA-approved drugs or licensed therapies on the market for hearing loss due to ototoxicity. As the field of inner ear therapeutics evolves, drug delivery strategies must recognize the relationships between therapeutic agents, formulations, delivery systems, and the disease. Treatment options for hearing loss will undeniably be further refined and optimized in the coming years as new therapeutics become available. Future research is needed to identify new mechanisms of action and delivery that will enable exciting novel treatments for inner ear disorders.

## Figures and Tables

**Table 1:** Studies Investigating the Administration of Nanoparticles in the Inner Ear.

	Administration Route	Evaluation of Uptake	References
**Natural Protein Nanoparticles**			
Bovine serum albumin nanoparticles	Transtympanic injection	Fluorescence microscopy and SEM	[80]
Poly (2-hydroxyethyl l-aspartamide; PHEA) nanoparticles	Transtympanic injection	Fluorescence microscopy	[81]
PHEA-g-C18-Arg8 (PCA) nanoparticles	In vitro	TEM	[82]
**Metallic Nanoparticles**			
Polyvinylpyrrolidone silver nanoparticles	Transtympanic injection	Micro CT imaging	[83]
Gold nanoparticles	Microbubbles and intratympanic injection	SEM, TEM, ABR, confocal microscopy and mass spectrometry	[84]
**Polymeric Nanoparticles**			
PLGA nanoparticles	Transtympanic injection	HPLC analysis	[85]
PLGA nanoparticles	Transtympanic injection	HPLC analysis	[86]
PLGA nanoparticles	In vitro	HPLC analysis	[87]
PEG-conjugated magnetic nanoparticles	Ex-vivo	Optical microscopy	[88]
**Inorganic Nanoparticles**			
SPION	Transtympanic and intracochlear injection	MRI and TEM	[89]
SPION	Organotypic culture	Light microscopy and TEM	[90]
SPION	Transtympanic injection	MRI	[91]
**Liposomes**			
Liposomes	Transtympanic injection	MRI	[92]
Liposomes	Drops to the tympanic membrane	Confocal microscopy	[93]
Liposomes	Intracochlear osmotic pump	MRI and cryo-TEM	[94]
Liposomes	In vitro	Confocal microscopy	[95]
**Lipid Nanoparticles**			
Solid lipid nanoparticles	Transtympanic injection	ABR and light microscopy	[96]
Solid lipid nanoparticles	In vitro	Confocal microscopy and flow cytometry	[97]
Solid lipid nanoparticles	Transtympanic injection	HPLC analysis	[98]
Phospholipid nanoparticles	Transtympanic injection	ABR and confocal imaging	[99]

## Data Availability

Data sharing not applicable.

## References

[1] Bernabei R., Bonuccelli U., Maggi S., Marengoni A., Martini A., Memo M., Pecorelli S., Peracino A.P., Quaranta N., Stella R. (2014). Hearing loss and cognitive decline in older adults: Questions and answers. Aging Clin. Exp. Res..

[2] Daniel E. (2007). Noise and hearing loss: A review. J. Sch. Health.

[3] Lin F.R., Yaffe K., Xia J., Xue Q.L., Harris T.B., Purchase-Helzner E., Satterfield S., Ayonayon H.N., Ferrucci L., Simonsick E.M. (2013). Hearing Loss and Cognitive Decline in Older Adults. JAMA Intern. Med..

[4] McCall A.A., Swan E.E.L., Borenstein J.T., Sewell W.F., Kujawa S.G., McKenna M.J. (2010). Drug delivery for treatment of inner ear disease: Current state of knowledge. Ear Hear..

[5] Kros C.J., Steyger P.S. (2019). Aminoglycoside- and Cisplatin-Induced Ototoxicity: Mechanisms and Otoprotective Strategies. Cold Spring Harb. Perspect. Med..

[6] Swan E.E.L., Mescher M.J., Sewell W.F., Tao S.L., Borenstein J.T. (2008). Inner ear drug delivery for auditory applications. Adv. Drug Deliv. Rev..

[7] Agrahari V., Agrahari V., Mitra A.K. (2017). Inner ear targeted drug delivery: What does the future hold?. Ther. Deliv..

[8] Van der Jeught S., Dirckx J.J.J., Aerts J.R.M., Bradu A., Podoleanu A.G., Buytaert J.A.N. (2013). Full-Field Thickness Distribution of Human Tympanic Membrane Obtained with Optical Coherence Tomography. J. Assoc. Res. Otolaryngol..

[9] Szymanski A., Toth J., Ogorevc M., Geiger Z. (2022). Anatomy, Head and Neck, Ear Tympanic Membrane.

[10] Gyo K., Aritomo H., Goode R.L. (1987). Measurement of the ossicular vibration ratio in human temporal bones by use of a video measuring system. Acta Oto Laryngol..

[11] Zdilla M.J., Skrzat J., Kozerska M., Leszczyński B., Tarasiuk J., Wroński S. (2018). Oval window size and shape: A micro-CT anatomical study with considerations for stapes surgery. Otol. Neurotol..

[12] Mancheño M., Aristegui M., Sañudo J.R. (2017). Round and Oval Window Anatomic Variability: Its Implication for the Vibroplasty Technique. Otol. Neurotol..

[13] Goycoolea M.V., Lundman L. (1997). Round window membrane. Structure function and permeability: A review. Microsc. Res. Tech..

[14] Liu H., Hao J., Li K.S. (2013). Current strategies for drug delivery to the inner ear. Acta Pharm. Sin. B.

[15] Zhang X., Gan R.Z. (2013). Dynamic Properties of Human Round Window Membrane in Auditory Frequencies. Med. Eng. Phys..

[16] Szeto B., Chiang H., Valentini C., Yu M., Kysar J.W., Lalwani A.K. (2020). Inner ear delivery: Challenges and opportunities. Laryngoscope Investig. Otolaryngol..

[17] Ren Y., Landegger L.D., Stankovic K.M. (2019). Gene therapy for human sensorineural hearing loss. Front. Cell. Neurosci..

[18] Peppi M., Marie A., Belline C., Borenstein J.T. (2018). Intracochlear drug delivery systems: A novel approach whose time has come. Expert Opin. Drug Deliv..

[19] Chae R., Rodriguez Rubio R. (2020). Anatomy of petrous face. Handb. Clin. Neurol..

[20] Sakamoto T., Hiraumi H. (2014). Anatomy of the inner ear. Regenerative Medicine for the Inner Ear.

[21] Nayak G., Lee S.I., Yousaf R., Edelmann S.E., Trincot C., van Itallie C.M., Sinha G.P., Rafeeq M., Jones S.M., Belyantseva I.A. (2013). Tricellulin deficiency affects tight junction architecture and cochlear hair cells. J. Clin. Investig..

[22] Hibino H., Nin F., Tsuzuki C., Kurachi Y. (2010). How is the highly positive endocochlear potential formed? The specific architecture of the *Stria vascularis* and the roles of the ion-transport apparatus. Pflug. Arch. Eur. J. Physiol..

[23] Echteler S.M., Fay R.R., Popper A.N., Fay R.R., Popper A.N. (1994). Structure of the mammalian cochlea. Comparative Hearing: Mammals.

[24] Nyberg S., Joan Abbott N., Shi X., Steyger P.S., Dabdoub A. (2019). Delivery of therapeutics to the inner ear: The challenge of the blood-labyrinth barrier. Sci. Transl. Med..

[25] Abbott N.J., Rönnbäck L., Hansson E. (2006). Astrocyte—Endothelial interactions at the blood–brain barrier. Nat. Rev. Neurosci..

[26] Koo J.W., Quintanilla-Dieck L., Jiang M., Liu J., Urdang Z.D., Allensworth J.J., Cross C.P., Li H., Steyger P.S. (2015). Endotoxemia-mediated inflammation potentiates aminoglycoside-induced ototoxicity. Sci. Transl. Med..

[27] Marcotti W., van Netten S.M., Kros C.J. (2005). The aminoglycoside antibiotic dihydrostreptomycin rapidly enters mouse outer hair cells through the mechano-electrical transducer channels. J. Physiol..

[28] Karasawa T., Wang Q., Fu Y., Cohen D.M., Steyger P.S. (2008). TRPV4 enhances the cellular uptake of aminoglycoside antibiotics. J. Cell Sci..

[29] Jiang M., Wang Q., Karasawa T., Koo J.W., Li H., Steyger P.S. (2014). Sodium-Glucose Transporter-2 (SGLT2; SLC5A2) Enhances Cellular Uptake of Aminoglycosides. PLoS ONE.

[30] Sojo-Dorado J., Rodríguez-Baño J., Grayson M.L., Cosgrove S.E., Crowe S., Hope W., McCarthy J.S., Mills J., Mouton J.W., Paterson D.L. (2022). Gentamicin. Kucer’s the Use of Antibiotics: A Clinical Review of Antibacterial, Antifungal, Antiparasitic, and Antiviral Drugs.

[31] Piu F., Bishop K.M. (2019). Local drug delivery for the treatment of neurotology disorders. Front. Cell. Neurosci..

[32] Hoskison E., Daniel M., Al-Zahid S., Shakesheff K.M., Bayston R., Birchall J.P. (2013). Drug delivery to the ear. Ther. Deliv..

[33] Wooltorton E. (2002). Health and Drug Alerts: Ototoxic effects from gentamicin ear drops. Can. Med. Assoc. J..

[34] Macfadyen C.A., Acuin J.M., Gamble C.L. (2005). Topical antibiotics without steroids for chronically discharging ears with underlying eardrum perforations. Cochrane Database Syst. Rev..

[35] Liu H., Feng L., Tolia G., Liddell M.R., Hao J., Li S.K. (2014). Evaluation of intratympanic formulations for inner ear delivery: Methodology and sustained release formulation testing. Drug Dev. Ind. Pharm..

[36] Li W., Hartsock J.J., Dai C., Salt A.N. (2018). Permeation Enhancers for Intratympanically-Applied Drugs studied using Fluorescent Dexamethasone as a Marker. Otol. Neurotol..

[37] Yoda S., Cureoglu S., Shimizu S., Morita N., Fukushima H., Sato T., Harada T., Paparella M.M. (2011). Round window membrane in Ménière’s disease: A human temporal bone study. Otol. Neurotol..

[38] King E.B., Salt A.N., Kel G.E., Eastwood H.T., O’Leary S.J. (2013). Gentamicin administration on the stapes footplate causes greater hearing loss and vestibulotoxicity than round window administration in guinea pigs. Hear. Res..

[39] King E.B., Shepherd R.K., Brown D.J., Fallon J.B. (2017). Gentamicin Applied to the Oval Window Suppresses Vestibular Function in Guinea Pigs. J. Assoc. Res. Otolaryngol..

[40] Cros O., Borga M., Pauwels E., Dirckx J.J.J., Gaihede M. (2013). Micro-channels in the mastoid anatomy. Indications of a separate blood supply of the air cell system mucosa by micro-CT scanning. Hear. Res..

[41] Gaihede M. (2015). Treatment of Otitis Media with Retroauricular Steroid Injection—Aalborg University’s Research Portal. https://vbn.aau.dk/en/publications/treatment-of-otitis-media-with-retroauricular-steroid-injection.

[42] Fooken Jensen P.V., Gaihede M. (2016). Congestion of mastoid mucosa and influence on middle ear pressure—Effect of retroauricular injection of adrenaline. Hear. Res..

[43] Chen D., Li Z., Zhou Q., Chen Y., Yang L., Tan J., Zeng X., Li P. (2020). Impacts of different methylprednisolone administration routes in patients with sudden hearing loss or Meniere’s disease. J. Otol..

[44] Chen A., Liu W., Xu L., Hou Z., Fan Z., Wang H., Wang M. (2022). Comparison of the Pathway to the Inner Ear Between Postauricular and Intramuscular Injection of Dexamethasone in Guinea Pigs. Front. Neurol..

[45] Plontke S.K., Hartsock J.J., Gill R.M., Salt A.N. (2016). Intracochlear Drug Injections through the Round Window Membrane: Measures to Improve Drug Retention. Audiol. Neurotol..

[46] Manrique-Huarte R., de Linera-Alperi M.A., Parilli D., Rodriguez J.A., Borro D., Dueck W.F., Smyth D., Salt A., Manrique M. (2021). Inner ear drug delivery through a cochlear implant: Pharmacokinetics in a Macaque experimental model. Hear. Res..

[47] Pararas E.E.L., Borkholder D.A., Borenstein J.T. (2012). Microsystems Technologies for Drug Delivery to the Inner Ear. Adv. Drug Deliv. Rev..

[48] Shimogori H., Yamashita H. (2000). Efficacy of intracochlear administration of betamethasone on peripheral vestibular disorder in the guinea pig. Neurosci. Lett..

[49] Abaamrane L., Raffin F., Schmerber S., Sendowski I. (2011). Intracochlear perfusion of leupeptin and z-VAD-FMK: Influence of antiapoptotic agents on gunshot-induced hearing loss. Eur. Arch. Oto-Rhino-Laryngol..

[50] Tandon V., Kang W.S., Robbins T.A., Spencer A.J., Kim E.S., McKenna M.J., Kujawa S.G., Fiering J., Pararas E.E.L., Mescher M.J. (2016). Microfabricated reciprocating micropump for intracochlear drug delivery with integrated drug/fluid storage and electronically controlled dosing. Lab Chip.

[51] Plontke S.K., Götze G., Rahne T., Liebau A. (2017). Intracochlear drug delivery in combination with cochlear implants: Current aspects. HNO.

[52] Boisvert I., Reis M., Au A., Cowan R., Dowell R.C. (2020). Cochlear implantation outcomes in adults: A scoping review. PLoS ONE.

[53] Benet L.Z., Hosey C.M., Ursu O., Oprea T.I. (2016). BDDCS, the Rule of 5 and drugability. Adv. Drug Deliv. Rev..

[54] Barich D.H., Zell M.T., Munson E.J., Wang B., Siahaan T.J., Soltero R. (2016). Physicochemical properties, formulation, and drug delivery. Drug Delivery: Principles and Applications.

[55] Lipinski C.A., Lombardo F., Dominy B.W., Feeney P.J. (2001). Experimental and computational approaches to estimate solubility and permeability in drug discovery and development settings. Adv. Drug Deliv. Rev..

[56] Doak B.C., Over B., Giordanetto F., Kihlberg J. (2014). Oral druggable space beyond the rule of 5: Insights from drugs and clinical candidates. Chem. Biol..

[57] Hao J., Li S.K. (2019). Inner ear drug delivery: Recent advances, challenges, and perspective. Eur. J. Pharm. Sci..

[58] Kanzaki S. (2018). Gene Delivery into the Inner Ear and Its Clinical Implications for Hearing and Balance. Molecules.

[59] Rathnam C., Chueng S.T.D., Ying YL M., Lee K.B., Kwan K. (2019). Developments in Bio-Inspired Nanomaterials for Therapeutic Delivery to Treat Hearing Loss. Front. Cell. Neurosci..

[60] Patel J., Szczupak M., Rajguru S., Balaban C., Hoffer M.E. (2019). Inner ear therapeutics: An overview of middle ear delivery. Front. Cell. Neurosci..

[61] Dumortier G., Grossiord J.L., Agnely F., Chaumeil J.C. (2006). A review of poloxamer 407 pharmaceutical and pharmacological characteristics. Pharm. Res..

[62] Chai Q., Jiao Y., Yu X. (2017). Hydrogels for Biomedical Applications: Their Characteristics and the Mechanisms behind Them. Gels.

[63] Fariba G., Farahani S.V. (2020). Theoretical Description of Hydrogel Swelling: A Review. Iran. Polym. J..

[64] El Kechai N., Agnely F., Mamelle E., Nguyen Y., Ferrary E., Bochot A. (2015). Recent advances in local drug delivery to the inner ear. Int. J. Pharm..

[65] Lajud S.A., Nagda D.A., Qiao P., Tanaka N., Civantos A., Gu R., Cheng Z., Tsourkas A., O’Malley B.W., Li D. (2015). A Novel Chitosan-Hydrogel-Based Nanoparticle Delivery System for Local Inner Ear Application. Otol. Neurotol..

[66] Hütten M., Dhanasingh A., Hessler R., Stöver T., Esser K.H., Möller M., Lenarz T., Jolly C., Groll J., Scheper V. (2014). In Vitro and In Vivo Evaluation of a Hydrogel Reservoir as a Continuous Drug Delivery System for Inner Ear Treatment. PLoS ONE.

[67] Gausterer J.C., Saidov N., Ahmadi N., Zhu C., Wirth M., Reznicek G., Arnoldner C., Gabor F., Honeder C. (2020). Intratympanic application of poloxamer 407 hydrogels results in sustained N-acetylcysteine delivery to the inner ear. Eur. J. Pharm. Biopharm..

[68] Borden R.C., Saunders J.E., Berryhill W.E., Krempl G.A., Thompson D.M., Queimado L. (2011). Hyaluronic Acid Hydrogel Sustains the Delivery of Dexamethasone across the Round Window Membrane. Audiol. Neurotol..

[69] Yu D., Sun C., Zheng Z., Wang X., Chen D., Wu H., Wang X., Shi F. (2016). Inner ear delivery of dexamethasone using injectable silk-polyethylene glycol (PEG) hydrogel. Int. J. Pharm..

[70] Shibata S.B., Cortez S.R., Wiler J.A., Swiderski D.L., Raphael Y. (2012). Hyaluronic Acid Enhances Gene Delivery into the Cochlea. Hum. Gene Ther..

[71] Giuliano E., Paolino D., Fresta M., Cosco D. (2018). Mucosal Applications of Poloxamer 407-Based Hydrogels: An Overview. Pharmaceutics.

[72] Fakhari A., Corcoran M., Schwarz A. (2017). Thermogelling properties of purified poloxamer 407. Heliyon.

[73] Russo E., Villa C. (2019). Poloxamer Hydrogels for Biomedical Applications. Pharmaceutics.

[74] Wang X., Dellamary L., Fernandez R., Harrop A., Keithley E.M., Harris J.P., Ye Q., Lichter J., Lebel C., Piu F. (2009). Dose-dependent sustained release of dexamethasone in inner ear cochlear fluids using a novel local delivery approach. Audiol. Neuro Otol..

[75] Dickey D.T., Muldoon L.L., Kraemer D.F., Neuwelt E.A. (2004). Protection against cisplatin-induced ototoxicity by N-acetylcysteine in a rat model. Hear. Res..

[76] Patra J.K., Das G., Fraceto L.F., Campos EV R., Rodriguez-Torres MD P., Acosta-Torres L.S., Diaz-Torres L.A., Grillo R., Swamy M.K., Sharma S. (2018). Nano based drug delivery systems: Recent developments and future prospects. J. Nanobiotechnology.

[77] Mirza A.Z., Siddiqui F.A., Mirza A.Z., Siddiqui F.A. (2014). Nanomedicine and drug delivery: A mini review. Int. Nano Lett..

[78] Lu H., Wang J., Wang T., Zhong J., Bao Y., Hao H. (2016). Recent Progress on Nanostructures for Drug Delivery Applications. J. Nanomater..

[79] Seymour L.W., Ulbrich K., Steyger P.S., Brereton M., Subr V., Strohalm J., Duncan R. (1994). Tumour tropism and anti-cancer efficacy of polymer-based doxorubicin prodrugs in the treatment of subcutaneous murine B16F10 melanoma. Br. J. Cancer.

[80] Yu Z., Yu M., Zhang Z., Hong G., Xiong Q. (2014). Bovine serum albumin nanoparticles as controlled release carrier for local drug delivery to the inner ear. Nanoscale Res. Lett..

[81] Yoon J.Y., Yang K.J., Kim D.E., Lee K.Y., Park S.N., Kim D.K., Kim J.D. (2015). Intratympanic delivery of oligoarginine-conjugated nanoparticles as a gene (or drug) carrier to the inner ear. Biomaterials.

[82] Yoon J.Y., Yang K.J., Park S.N., Kim D.K., Kim J.D. (2016). The effect of dexamethasone/cell-penetrating peptide nanoparticles on gene delivery for inner ear therapy. Int. J. Nanomed..

[83] Zou J., Hannula M., Misra S., Feng H., Labrador R.H., Aula A.S., Hyttinen J., Pyykkö I. (2015). Micro CT visualization of silver nanoparticles in the middle and inner ear of rat and transportation pathway after transtympanic injection. J. Nanobiotechnol..

[84] Lin Y.C., Shih C.P., Chen H.C., Chou Y.L., Sytwu H.K., Fang M.C., Lin Y.Y., Kuo C.Y., Su H.H., Hung C.L. (2021). Ultrasound Microbubble–Facilitated Inner Ear Delivery of Gold Nanoparticles Involves Transient Disruption of the Tight Junction Barrier in the Round Window Membrane. Front. Pharmacol..

[85] Zhang X., Chen G., Wen L., Yang F., Shao A.L., Li X., Long W., Mu L. (2013). Novel multiple agents loaded PLGA nanoparticles for brain delivery via inner ear administration: In vitro and in vivo evaluation. Eur. J. Pharm. Sci..

[86] Cai H., Wen X., Wen L., Tirelli N., Zhang X., Zhang Y., Su H., Yang F., Chen G. (2014). Enhanced local bioavailability of single or compound drugs delivery to the inner ear through application of PLGA nanoparticles via round window administration. Int. J. Nanomed..

[87] Kim D.-H., Nguyen T.N., Han Y.-M., Tran P., Rho J., Lee J.-Y., Son H.-Y., Park J.-S. (2021). Local drug delivery using poly(lactic-co-glycolic acid) nanoparticles in thermosensitive gels for inner ear disease treatment. Drug Deliv..

[88] Lee J.H., Kim J.W., Levy M., Kao A., Noh S.H., Bozovic D., Cheon J. (2014). Magnetic nanoparticles for ultrafast mechanical control of inner ear hair cells. ACS Nano.

[89] Zou J., Zhang W., Poe D., Qin J., Fornara A., Zhang Y., Ramadan U.A., Muhammed M., Pyykkö I. (2010). MRI manifestation of novel superparamagnetic iron oxide nanoparticles in the rat inner ear. Nanomedicine.

[90] Thaler M., Roy S., Fornara A., Bitsche M., Qin J., Muhammed M., Salvenmoser W., Rieger G., Fischer A.S., Glueckert R. (2011). Visualization and analysis of superparamagnetic iron oxide nanoparticles in the inner ear by light microscopy and energy filtered TEM. Nanomed. Nanotechnol. Biol. Med..

[91] Zou J., Ostrovsky S., Israel L.L., Feng H., Kettunen M.I., Lellouche J.P.M., Pyykkö I. (2017). Efficient penetration of ceric ammonium nitrate oxidant-stabilized gamma-maghemite nanoparticles through the oval and round windows into the rat inner ear as demonstrated by MRI. J. Biomed. Mater. Res. Part B Appl. Biomater..

[92] Zou J., Sood R., Ranjan S., Poe D., Ramadan U.A., Kinnunen P.K.J., Pyykkö I. (2010). Manufacturing and in vivo inner ear visualization of MRI traceable liposome nanoparticles encapsulating gadolinium. J. Nanobiotechnol..

[93] Sadabad R.K., Xia A., Benkafadar N., Faniku C., Preciado D., Yang S., Valdez T.A. (2022). Topical Delivery of Elastic Liposomal Vesicles for Treatment of Middle and Inner Ear Disease. bioRxiv.

[94] Zou J., Sood R., Zhang Y., Kinnunen P.K.J., Pyykkö I. (2014). Pathway and morphological transformation of liposome nanocarriers after release from a novel sustained inner-ear delivery system. Nanomedicine.

[95] Curcio M., Cirillo G., Amato R., Guidotti L., Amantea D., de Luca M., Nicoletta F.P., Iemma F., Garcia-Gil M. (2022). Encapsulation of Alpha-Lipoic Acid in Functional Hybrid Liposomes: Promising Tool for the Reduction of Cisplatin-Induced Ototoxicity. Pharmaceuticals.

[96] Gao W., Zhang Y., Zhang Q., Zhang L. (2016). Nanoparticle-Hydrogel: A Hybrid Biomaterial System for Localized Drug Delivery. Ann. Biomed. Eng..

[97] Cervantes B., Arana L., Murillo-Cuesta S., Bruno M., Alkorta I., Varela-Nieto I. (2019). Solid Lipid Nanoparticles Loaded with Glucocorticoids Protect Auditory Cells from Cisplatin-Induced Ototoxicity. J. Clin. Med..

[98] Chen G., Hou S.-X., Hu P. (2008). In vitro dexamethasone release from nanoparticles and its pharmacokinetics in the inner ear after administration of the drug-loaded nanoparticles via the round window. Nan Fang Yi Ke Da Xue Xue Bao.

[99] Yang K.J., Son J., Jung S.Y., Yi G., Yoo J., Kim D.K., Koo H. (2018). Optimized phospholipid-based nanoparticles for inner ear drug delivery and therapy. Biomaterials.

[100] Pyykko I., Zou J., Zhang Y., Zhang W., Feng H., Kinnunen P. (2013). Nanoparticle based inner ear therapy. World J. Otorhinolaryngol..

[101] Zhang L., Xu Y., Cao W., Xie S., Wen L., Chen G. (2018). Understanding the translocation mechanism of PLGA nanoparticles across round window membrane into the inner ear: A guideline for inner ear drug delivery based on nanomedicine. Int. J. Nanomed..

[102] Scioli Montoto S., Muraca G., Ruiz M.E. (2020). Solid Lipid Nanoparticles for Drug Delivery: Pharmacological and Biopharmaceutical Aspects. Front. Mol. Biosci..

[103] Huynh N.T., Passirani C., Saulnier P., Benoit J.P. (2009). Lipid nanocapsules: A new platform for nanomedicine. Int. J. Pharm..

[104] Li L., Chao T., Brant J., O’Malley B., Tsourkas A., Li D. (2017). Advances in Nano-based Inner Ear Delivery Systems for the Treatment of Sensorineural Hearing Loss. Adv. Drug Deliv. Rev..

[105] Scheper V., Wolf M., Scholl M., Kadlecova Z., Perrier T., Klok H.A., Saulnier P., Lenarz T., Stöver T. (2009). Potential novel drug carriers for inner ear treatment: Hyperbranched polylysine and lipid nanocapsules. Nanomedicine.

[106] Mittal R., Pena S.A., Zhu A., Eshraghi N., Fesharaki A., Horesh E.J., Mittal J., Eshraghi A.A. (2019). Nanoparticle-based drug delivery in the inner ear: Current challenges, limitations and opportunities. Artif. Cells Nanomed. Biotechnol..

[107] Wareing M., Mhatre A.N., Pettis R., Han J.J., Haut T., Pfister M.H.F., Hong K., Zheng W.W., Lalwani A.K. (1999). Cationic liposome mediated transgene expression in the guinea pig cochlea. Hear. Res..

[108] Okano T., Nakagawa T., Kita T., Endo T., Ito J. (2006). Cell-gene delivery of brain-derived neurotrophic factor to the mouse inner ear. Mol. Ther..

[109] Ge X., Jackson R.L., Liu J., Harper E.A., Hoffer M.E., Wassel R.A., Dormer K.J., Kopke R.D., Balough B.J. (2007). Distribution of PLGA nanoparticles in chinchilla cochleae. Otolaryngol. Head Neck Surg..

[110] Shimoji M., Ramaswamy B., Shukoor M.I., Benhal P., Broda A., Kulkarni S., Malik P., McCaffrey B., Lafond J.F., Nacev A. (2019). (2019). Toxicology study for magnetic injection of prednisolone into the rat cochlea. Eur. J. Pharm. Sci..

[111] Avasthi A., Caro C., Pozo-Torres E., Leal M.P., García-Martín M.L. (2020). Magnetic Nanoparticles as MRI Contrast Agents. Top. Curr. Chem..

[112] Kopke R.D., Wassel R.A., Mondalek F., Grady B., Chen K., Liu J., Gibson D., Dormer K.J. (2006). Magnetic Nanoparticles: Inner Ear Targeted Molecule Delivery and Middle Ear Implant. Audiol. Neurotol..

[113] Chen G., Zhang X., Yang F., Mu L. (2010). Disposition of nanoparticle-based delivery system via inner ear administration. Curr. Drug Metab..

[114] Buckiová D., Ranjan S., Newman T.A., Johnston A.H., Sood R., Kinnunen P.K.J., Popelář J., Chumak T., Syka J. (2012). Minimally invasive drug delivery to the cochlea through application of nanoparticles to the round window membrane. Nanomedicine.

[115] Zou J., Saulnier P., Perrier T., Zhang Y., Manninen T., Toppila E., Pyykkö I. (2008). Distribution of lipid nanocapsules in different cochlear cell populations after round window membrane permeation. J. Biomed. Mater. Res. Part B Appl. Biomater..

[116] Wen X., Ding S., Cai H., Wang J., Wen L., Yang F., Chen G. (2016). Nanomedicine strategy for optimizing delivery to outer hair cells by surface-modified poly(lactic/glycolic acid) nanoparticles with hydrophilic molecules. Int. J. Nanomed..

[117] Singh N., Jenkins G.J.S., Asadi R., Doak S.H. (2010). Potential toxicity of superparamagnetic iron oxide nanoparticles (SPION). Nano Rev..

[118] Musazzi U.M., Franzé S., Cilurzo F. (2018). Innovative pharmaceutical approaches for the management of inner ear disorders. Drug Deliv. Transl. Res..

[119] Lee J.J., Jang J.H., Choo O.S., Lim H.J., Choung Y.H. (2018). Steroid intracochlear distribution differs by administration method: Systemic versus intratympanic injection. Laryngoscope.

[120] Fröhlich E. (2012). The role of surface charge in cellular uptake and cytotoxicity of medical nanoparticles. Int. J. Nanomed..

[121] Young C.C., Vedadghavami A., Bajpayee A.G. (2020). Bioelectricity for Drug Delivery: The Promise of Cationic Therapeutics. Bioelectricity.

[122] Nomura Y. (1984). Otological significance of the round window. Adv. Otorhinolaryngol..

[123] Zhang R., Qin X., Kong F., Chen P., Pan G. (2019). Improving cellular uptake of therapeutic entities through interaction with components of cell membrane. Drug Deliv..

[124] Derakhshankhah H., Jafari S. (2018). Cell penetrating peptides: A concise review with emphasis on biomedical applications. Biomed. Pharmacother..

[125] Wei X., Shao B., He Z., Ye T., Luo M., Sang Y., Liang X., Wang W., Luo S., Yang S. (2015). Cationic nanocarriers induce cell necrosis through impairment of Na^+^/K^+^-ATPase and cause subsequent inflammatory response. Cell Res..

[126] Lv H., Zhang S., Wang B., Cui S., Yan J. (2006). Toxicity of cationic lipids and cationic polymers in gene delivery. J. Control. Release.

[127] Zhou H., Ma X., Liu Y., Dong L., Luo Y., Zhu G., Qian X., Chen J., Lu L., Wang J. (2015). Linear polyethylenimine-plasmid DNA nanoparticles are ototoxic to the cultured sensory epithelium of neonatal mice. Mol. Med. Rep..

[128] Cai H., Liang Z., Huang W., Wen L., Chen G. (2017). Engineering PLGA nano-based systems through understanding the influence of nanoparticle properties and cell-penetrating peptides for cochlear drug delivery. Int. J. Pharm..

[129] Dash-Wagh S., Jacob S., Lindberg S., Fridberger A., Langel Ü., Ulfendahl M. (2012). Intracellular Delivery of Short Interfering RNA in Rat Organ of Corti Using a Cell-penetrating Peptide PepFect6. Mol. Ther. Nucleic Acids.

[130] Youm I., Musazzi U.M., Gratton M.A., Murowchick J.B., Youan B.B.C. (2016). Label-Free Ferrocene-Loaded Nanocarrier Engineering for In Vivo Cochlear Drug Delivery and Imaging. J. Pharm. Sci..

[131] Iwai K., Nakagawa T., Endo T., Matsuoka Y., Kita T., Kim T.S., Tabata Y., Ito J. (2006). Cochlear protection by local insulin-like growth factor-1 application using biodegradable hydrogel. Laryngoscope.

[132] Xu X., Lin K., Wang Y., Xu K., Sun Y., Yang X., Yang M., He Z., Zhang Y., Zheng H. (2020). A metal–Organic framework based inner ear delivery system for the treatment of noise-induced hearing loss. Nanoscale.

[133] Milton Harris J., Chess R.B. (2003). Effect of pegylation on pharmaceuticals. Nat. Rev. Drug Discov..

[134] Salt A.N., Hirose K. (2018). Communication pathways to and from the inner ear and their contributions to drug delivery. Hear. Res..

[135] Salt A.N., Plontke S.K. (2018). Pharmacokinetic principles in the inner ear: Influence of drug properties on intratympanic applications. Hear. Res..

[136] King E.B., Salt A.N., Eastwood H.T., O’Leary S.J. (2011). Direct entry of gadolinium into the vestibule following intratympanic applications in Guinea pigs and the influence of cochlear implantation. J. Assoc. Res. Otolaryngol..

[137] Rybak L.P., Dhukhwa A., Mukherjea D., Ramkumar V. (2019). Local Drug Delivery for Prevention of Hearing Loss. Front. Cell. Neurosci..

[138] El Kechai N., Mamelle E., Nguyen Y., Huang N., Nicolas V., Chaminade P., Yen-Nicolaÿ S., Gueutin C., Granger B., Ferrary E. (2016). Hyaluronic acid liposomal gel sustains delivery of a corticoid to the inner ear. J. Control. Release.

[139] Salt A.N., Plontke S.K. (2009). Principles of Local Drug Delivery to the Inner Ear. Audiol. Neurotol..

[140] Guan L., Yang H., Cai Y., Sun L., Di P., Li W., Liu G., Tang Y. (2019). ADMET-score—A comprehensive scoring function for evaluation of chemical drug-likeness. MedChemComm.

[141] Abughazaleh R.D., Tracy T.S. (2014). Therapeutic Index. Wiley StatsRef Stat. Ref. Online.

[142] Sawamura S., Ogata G., Asai K., Razvina O., Ota T., Zhang Q., Madhurantakam S., Akiyama K., Ino D., Kanzaki S. (2021). Analysis of Pharmacokinetics in the Cochlea of the Inner Ear. Front. Pharmacol..

[143] Parnes L.S., Sun A.H., Freeman D.J. (1999). Corticosteroid pharmacokinetics in the inner ear fluids: An animal study followed by clinical application. Laryngoscope.

[144] Plontke S.K., Biegner T., Kammerer B., Delabar U., Salt A.N. (2008). Dexamethasone concentration gradients along scala tympani after application to the round window membrane. Otol. Neurotol..

[145] Hellberg V., Wallin I., Ehrsson H., Laurell G. (2013). Cochlear pharmacokinetics of cisplatin: An in vivo study in the guinea pig. Laryngoscope.

[146] Wang Y., Han L., Diao T., Jing Y., Wang L., Zheng H., Ma X., Qi J., Yu L. (2018). A comparison of systemic and local dexamethasone administration: From perilymph/cochlea concentration to cochlear distribution. Hear. Res..

[147] Grondin Y., Cotanche D.A., Manneberg O., Molina R., Treviño-Villarreal J.H., Sepulveda R., Clifford R., Bortoni M.E., Forsberg S., LaBrecque B. (2013). Pulmonary delivery of d-methionine is associated with an increase in ALCAR and glutathione in cochlear fluids. Hear. Res..

[148] Taylor A.E., Keevil B., Huhtaniemi I.T. (2015). Mass spectrometry and immunoassay: How to measure steroid hormones today and tomorrow. Eur. J. Endocrinol..

[149] Plontke S.K., Mynatt R., Gill R.M., Borgmann S., Salt A.N. (2007). Concentration gradient along the scala tympani after local application of gentamicin to the round window membrane. Laryngoscope.

[150] Hahn H., Salt A.N., Schumacher U., Plontke S.K. (2013). Gentamicin concentration gradients in scala tympani perilymph following systemic applications. Audiol. Neuro Otol..

[151] Roehm P., Hoffer M., Balaban C.D. (2007). Gentamicin uptake in the chinchilla inner ear. Hear. Res..

[152] Huisken J., Stainier D.Y.R. (2009). Selective plane illumination microscopy techniques in developmental biology. Development.

[153] McDonnell L.A., Heeren R.M.A. (2007). Imaging mass spectrometry. Mass Spectrom. Rev..

[154] Bagotsky V.S. (2006). Fundamentals of Electrochemistry.

[155] Jackowska K., Krysinski P. (2013). New trends in the electrochemical sensing of dopamine. Anal. Bioanal. Chem..

[156] Liu J., Yu P., Lin Y., Zhou N., Li T., Ma F., Mao L. (2012). In vivo electrochemical monitoring of the change of cochlear perilymph ascorbate during salicylate-induced tinnitus. Anal. Chem..

